# Association of long-term quality of life, safety, and efficacy: robotic vs. open thyroidectomy for benign thyroid nodules

**DOI:** 10.1097/JS9.0000000000003540

**Published:** 2025-09-24

**Authors:** Xiangquan Qin, Xia Liu, Yi Zhang

**Affiliations:** a Department of Breast and Thyroid Surgery, The Southwest Hospital of Army Military Medical University, Chongqing, China; b Department of Anesthesiology, the Southwest Hospital of Army Military Medical University, Chongqing, China.

**Keywords:** benign thyroid nodules, hypoparathyroidism, quality of life, robot surgery, thyroidectomy

## Abstract

**Objective:**

To compare the safety, efficacy, and quality of life (QoL) of robotic thyroidectomy (RT) and open thyroidectomy (OT) for benign thyroid nodules.

**Background:**

Traditional OT lacks aesthetic advantages, while endoscopic thyroidectomy has limitations for larger nodules. Advances in RT provide a minimally invasive option, offering improved aesthetics and efficacy for larger benign thyroid nodules.

**Methods:**

This retrospective cohort study included 1614 benign thyroid nodules patients from 2014 to 2024. Following propensity score matching (1:2 ratio), 1079 patients who underwent OT or RT were analyzed. Postoperative complications were recorded, and patients were assessed using QoL and aesthetic outcome scales.

**Results:**

In the OT group, 186 patients (25.34%) had inadvertent parathyroid resection vs. none in the RT group (*P* < 0.001). Transient hypoparathyroidism affected 129 OT patients (17.57%) vs. 36 RT patients (10.43%), respectively (*P* = 0.002). Swallowing Impairment Score and Voice Impairment Score were higher in the OT group after 5 years (*P* < 0.001). The RT group showed better “general health” and “vitality” at 0.5–3 years and >5 years, and better “social function,” “mental health,” and “role emotional” at >5 years (*P* < 0.05). Scar lengths were shorter in the RT group at all time points (*P* < 0.001), while SCAR-Q appearance scores were higher in the OT group (*P* < 0.001).

**Conclusion:**

Robotic thyroidectomy demonstrated superior cosmetic outcomes, lower complication rates, and minimal impact on postoperative QoL compared to open surgery for benign thyroid nodules, including larger ones. Therefore, with appropriate patient selection, robotic thyroidectomy may be the preferred approach for benign thyroid disease.

**HIGHLIGHTS**

- It was a large-scale and long-term observational clinical study focusing on thyroidectomy for benign thyroid disease, which holds significance as a reference for minimally invasive benign thyroidectomy.
- We conducted the first follow-up assessment of patients undergoing robotic thyroidectomy (RT) for benign tumors’ postoperative aesthetics, psychological effects, and quality of life. This contributes to a more comprehensive evaluation of the long-term effects of surgery and the overall health status of the patients.
- This was the first report of RT for larger thyroid disease, addressing the previous limitation of minimally invasive surgery in achieving resection of large masses and providing significant empirical support for technological advancements in this field.

## Introduction

Surgical treatment for thyroid cancer has received significant attention in the field of thyroid surgery^[1,2]^.While much of the focus has been on malignancy, such as hyperthyroidism, benign nodular goiter, benign thyroid nodules, and Hashimoto’s thyroiditis, are also critical aspects of thyroid surgical management due to their high prevalence rates.^[3–5]^ Traditional open thyroidectomy (OT) often results in visible scars on the front of the neck, influencing the aesthetic appearance of the neck[6]. Aesthetic outcomes are crucial for surgical patients, especially in visible areas like the neck[7]. Avoiding neck scars is essential for patient satisfaction and psychological well-being[8]. Endoscopic thyroidectomy through concealed approaches can prevent neck disfigurement, but the long straight instruments used in this approach lack the flexibility offered by manual dexterity in open surgery[9]. A literature review^[10–12]^ found that it is still hindered by inherent limitations such as limited range of motion, physiological tremor amplification, difficulty in suturing at certain sites, and a steep learning curve. Moreover, benign thyroid nodules, particularly those with larger diameters(≥5 cm), tend to have rich blood supplies, which increases the risk of intraoperative bleeding and makes bleeding control more challenging^[13,14]^. Consequently, there are strict criteria for selecting candidates for endoscopic thyroidectomy for larger benign nodules^[15,16]^.

Endo-wrist instruments (Intuitive Surgical, Sunnyvale, California, USA) used in robotic surgery offer greater flexibility and precision than traditional endoscopic instruments, achieving levels of skill that may surpass manual capabilities^[17,18]^. Therefore, robotic surgery theoretically has broader indications than endoscopic surgery. Although literature exists on the advantages of robotic thyroidectomy for benign thyroid disease[19], there are currently no comparative studies evaluating the safety, efficacy, and quality of life (QoL) between robotic thyroidectomy and open surgery for benign thyroid nodules, especially for larger ones, in studies with long-term follow-up and large sample sizes. Therefore, we retrospectively analyzed data from patients who underwent RT or OT for benign nodules at our hospital over the past 10 years between 2014 and 2024.

## Methods

**Figure 1.**
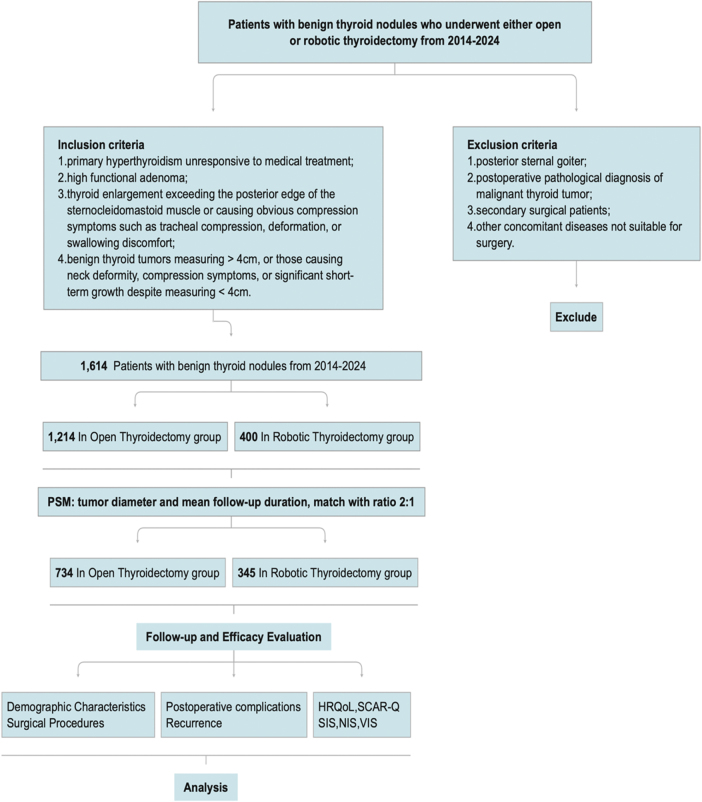
Flowchart of the study protocol.

The work has been reported in line with the STROCSS criteria[20]. Given the retrospective nature of the data, the requirement for patient informed consent was waived. We conducted a retrospective analysis of surgical data from patients with benign thyroid nodules who underwent thyroidectomy at our hospital between July 2014 and January 2024 (Fig. 1).

To minimize selection bias related to surgical proficiency, only cases from highly experienced surgical teams were included (OT: >500 cases/year, RT: >300 cases/year). The patients included in this study were selected based on the following criteria. All robotic thyroidectomy for large benign thyroid nodules employs either the UABA (Unilateral Axillo-Breast Approach)[21] or BABA (Bilateral Axillo-Breast Approach)[22], particularly for benign nodules larger than 5 cm, with a preference for the BABA approach^[23,24]^. Inclusion criteria[25]: (1) primary hyperthyroidism unresponsive to medical treatment; (2) high functional adenoma; (3) thyroid enlargement exceeding the posterior edge of the sternocleidomastoid muscle or causing obvious compression symptoms such as tracheal compression, deformation, or swallowing discomfort; and (4) benign thyroid tumors measuring >4 cm, or those causing neck deformity, compression symptoms, or significant short-term growth despite measuring <4 cm. The exclusion criteria were: (1) posterior sternal goiter; (2) postoperative pathological diagnosis of malignant thyroid tumor; (3) secondary surgical patients; and (4) other concomitant diseases unsuitable for surgery.

Additionally, all patients opted for RT or OT based on their preference. RT surgeries were performed using the Da Vinci S, Si, or Xi surgical system (Intuitive Surgical, Sunnyvale, California, USA)[26]. All patients completed evaluations using the Short Form (SF)-36 scale: physiological function, role physical function, body pain, general health, vitality, social function, role emotional, health change and mental health,[27] Voice Impairment Score (VIS),[28] Swallowing Impairment Score (SIS), Neck Impairment Score (NIS)[29], and Scar questionnaire (SCAR-Q) scale^[30,31]^. Patients were evaluated 0.5–3, 3–5, and >5 years after surgery to capture early recovery, postoperative complications, short-term outcomes, medium-term outcomes, and recovery stabilization, and long-term effects, respectively[32]. Ultimately, the loss to follow-up rate was controlled within 15%, which we believe is acceptable for surgical outcome studies. The surgical procedures, postoperative group comparisons, and relevant videos are provided as online Supplementary Digital Content eMethods, available at: http://links.lww.com/JS9/F210.

### Complications

We documented any episodes of tetany, transient, and permanent hypoparathyroidism[33], temporary and permanent hoarseness^[34,35]^, postoperative infection[36], and postoperative bleeding[37].

### Statistical analysis

Continuous variables are summarized as means and standard deviations, while categorical variables are presented as frequencies and proportions. The SF-36, NIS, VIS, SIS, and SCAR-Q data were transformed into total scale scores. Statistical analysis was performed using IBM SPSS Statistics for Macintosh, Version 29.0 (IBM Corp., Armonk, NY, USA). Figures were generated using Photoshop 2023 (Adobe, San Jose, CA, USA). We performed propensity score matching (PSM) analysis using the “MatchIt” package in R. The matching was based on multiple covariates, including the Sonographic tumor diameter and follow-up duration. We used the “Matchit()” function to perform matching, applying 1:2 nearest neighbor matching without a caliper, and calculated the propensity scores using logistic regression analysis. Statistical significance was determined using appropriate tests, with significance set at *P* < 0.05. An independent samples *t*-test was utilized to compare the means between two groups, while a chi-square test of independence was employed to examine the association between categorical variables.

## Results

### Baseline characteristics before and after propensity score matching

A total of 1614 patients participated in the study, with 1214 in the OT group and 400 in the RT group. PSM was conducted for mass sonographic tumor diameter and follow-up duration at a 2:1 ratio, including 1079 patients in the study. Ultimately, 345 patients in the RT group and 734 patients in the OT group agreed to participate and underwent postoperative follow-up assessments (Table 1).

**Table 1:** Baseline characteristics of patients before and after propensity score matching (PSM)

	Before PSM, *N* (%)			After PSM, *N* (%)		
	OT group (*N* = 1214)	RT group (*N* = 400)	*P*	OT group (*N* = 734)	RT group (*N* = 345)	*P*
Sonographic tumor diameter [median (min, max)/mm]	39.00 (2.80,112.00)	33.65 (3.70,93.00)	<0.001	37.95 (6.50,112.00)	35.00 (10.30,93.00)	0.34
Median follow-up (months)	74.00 (8.00, 122.00)	74.00 (8.00,122.00)	0.86	74.00 (8.00, 122.00)	74.00 (8.00,122.00)	0.74

OT, open thyroidectomy; RT, robotic thyroidectomy.

After patient screening, the mean age was 49.03 ± 11.74 years in the OT group and 41.93 ± 12.88 years in the RT group, exhibiting a significant difference (*P* < 0.001). Body mass index (BMI) was also significantly different between the two groups (*P* < 0.001), with median BMIs of 23.20 kg/m^2^ in the OT group and 22.35 kg/m^2^ in the RT group. There was no statistically significant difference in sonographic tumor size between the OT and RT groups (*P* > 0.05). Tumor sizes of 0–5 cm were observed in 615 patients (83.79%) in the OT group and 299 patients (86.67%) in the RT group. Tumor sizes of 5–6 cm were reported in 92 patients (12.53%) in the OT group and 35 (10.14%) in the RT group. For tumors >6 cm, 27 patients (3.68%) were in the OT group and 11 (3.19%) in the RT group. The maximum tumor sizes recorded were 11.2 cm in the OT group and 9.3 cm in the RT group. The average operation time for lobectomy procedures was 95.58 ± 36.26 min in the OT group and 127.28 ± 45.39 min in the RT group. For total thyroidectomy procedures, the average operation times were 111.32 ± 41.82 min and 135.51 ± 51.66 min, respectively, exhibiting significant differences (*P* < 0.001). In the OT group, 186 patients (25.34%) underwent inadvertent parathyroid gland resection, all of whom required parathyroid gland transplantation. In contrast, no cases of inadvertent parathyroid gland resection occurred in the RT group (*P* < 0.001). The median blood loss was 50 ml for OT group versus 30 ml for RT group, with a maximum blood loss of 800 ml observed in the OT group (*P* < 0.001).There were no significant differences in sex, ethnicity, marital status, comorbidity, tumor location, Hashimoto’s thyroiditis, drain insertion days, and median follow-up duration between the two groups (*P* > 0.05) (Table 2).

**Table 2:** Patient demographic characteristics, surgical procedures, and perioperative characteristics

	OT group (*N* = 734)	RT group (*N* = 345)	*P*
Age, years	49.03 ± 11.74	41.93 ± 12.88	<0.001
Sex			0.49
Female	637 (86.78)	294 (85.22)	
Male	97 (13.22)	51 (14.78)	
Ethnicity			0.33
Han	707 (96.32)	328 (95.07)	
Other	27 (3.68)	17 (4.93)	
Marital status			0.45
Unmarried	121 (16.48)	49 (14.20)	
Married	602 (82.02)	288 (83.48)	
Divorced	6 (0.82)	6 (1.74)	
Widow	5 (0.68)	2 (0.58)	
Body mass index [median (min, max)/kg/m^2^]	23.20 (14.57, 32.25)	22.35 (16.42, 33.71)	<0.001
Comorbidity			
Hypertension	53 (7.22)	31 (8.99)	0.31
Diabetes mellitus	23 (3.13)	10 (2.90)	0.83
Sonographic tumor size, cm/*N* (%)			0.45
> 0, <4	413 (56.27)	211 (61.16)	
≥4, <5	202 (27.52)	88 (25.51)	
≥5, <6	92 (12.53)	35 (10.14)	
≥6	27 (3.68)	11 (3.19)	
Tumor location			0.58
Unilateral	475 (64.72)	212 (61.45)	
Bilateral	257 (35.01)	132 38.26)	
Isthmus	2 (0.27)	1 (0.29)	
Hashimoto’s thyroiditis			0.12
Yes	29 (3.95)	21 (6.09)	
No	705 (96.05)	324 (93.91)	
Operative time (min)	98.47 ± 36.96	136.13 ± 46.88	<0.001
Lobectomy	95.58 ± 36.26	127.28 ± 45.39	<0.001
Total thyroidectomy	111.32 ± 41.82	135.51 ± 51.66	<0.001
Operation type, *N* (%)			0.28
Lobectomy	474 (64.58)	211 (61.16)	
Total thyroidectomy	260 (35.42)	134 (38.84)	
Inadvertent parathyroid gland resection			<0.001
Yes	186 (25.34)	0	
No	548 (74.66)	345 (100.00)	
Number of parathyroid glands transplanted, *N* (%)			<0.001
0	548 (74.66)	345 (100.00)	
1	164 (22.34)	0	
2	20 (2.72)	0	
3	2 (0.27)	0	
Intraoperative bleeding [median (min, max)/ml]	50.00 (5,800)	30.00 (3,350)	<0.001
Number of days of drain insertion, days	4.03 ± 1.19	4.23 ± 1.14	0.01

OT, open thyroidectomy; RT, robotic thyroidectomy.

Data are means ± SD, number (%), median (min, max)

### Complications

The rates of transient hypoparathyroidism were 129 (17.57%) vs. 36 (10.43%) in the OT and RT groups, respectively (*P* = 0.002). Compared to the OT group, the RT group exhibited lower rates of tetany 1 (0.29%) vs. 76 (10.35%), respectively (*P* < 0.001). Additionally, there were no cases of recurrence or permanent hoarseness in either group. There were no significant differences between the two groups in terms of postoperative bleeding, hematoma, lymphatic leakage, postoperative hypocalcemia, permanent hypoparathyroidism, or postoperative infection (*P* > 0.05) (Table 3).

**Table 3:** Postoperative complications

	OT group (*N* = 734)	RT group (*N* = 345)	*P*
Postoperative bleeding^c^	2 (0.27)	1 (0.29)	0.96
Hematoma (observed)	0	1 (0.29)	0.14
Lymphatic leakage	1 (0.14)	0	0.49
Tetany	76 (10.35)	1 (0.29)	<0.001
Postoperative hypocalcemia	262 (35.69)	135 (39.13)	0.51
Transient hypoparathyroidism^a^	129 (17.57)	36 (10.43)	0.002
Permanent hypoparathyroidism^a^	6 (0.82)	0	0.09
Permanent hoarseness^b^	0	0	1
Postoperative infection	0	1 (0.29)	0.14
Recurrence	0	0	1

OT, open thyroidectomy; RT, robotic thyroidectomy.

Data are presented as numbers (%).

^a^ Transient hypoparathyroidism was defined as serum PTH level falling below the normal range on the first day after surgery. If the PTH level fails to recover within 6 months after surgery, it was considered permanent hypoparathyroidism.

^b^ Temporary hoarseness refers to voice change 6 months after surgery due to recurrent laryngeal nerve injury. If hoarseness does not fully recover within 6 months after surgery, and is confirmed by laryngoscopy as vocal-cord paralysis, it was considered permanent recurrent laryngeal nerve injury.

^c^ Postoperative bleeding refers to bleeding in the original surgical field or subcutaneous tunnel area requiring further surgical intervention for bleeding control or to clear any subcutaneous hematomas.

### QoL assessment

#### Impairment

The OT group had higher SIS and VIS scores compared to the RT group at >5 years after surgery (*P* < 0.001). There were no differences between the two groups in the postoperative NIS, SIS, and VIS assessments at 0.5–3 years and 3–5 years(*P* > 0.05).

#### Cosmetic results

In the postoperative assessments at 1–3 years, 3–5 years, and >5 years, the RT group exhibited consistently shorter scar lengths compared to the OT group (*P* < 0.001). The SCAR-Q appearance score was significantly higher in the OT group compared to the RT group (14.51 ± 12.00 vs. 2.67 ± 7.78 points, *P* < 0.001).

Furthermore, there were no significant differences between the two groups in terms of scar-related symptoms and postoperative psychological effects at 3–5 years and >5 years (*P* > 0.05)

### HR-QoL (SF-36)

**Figure 2.**
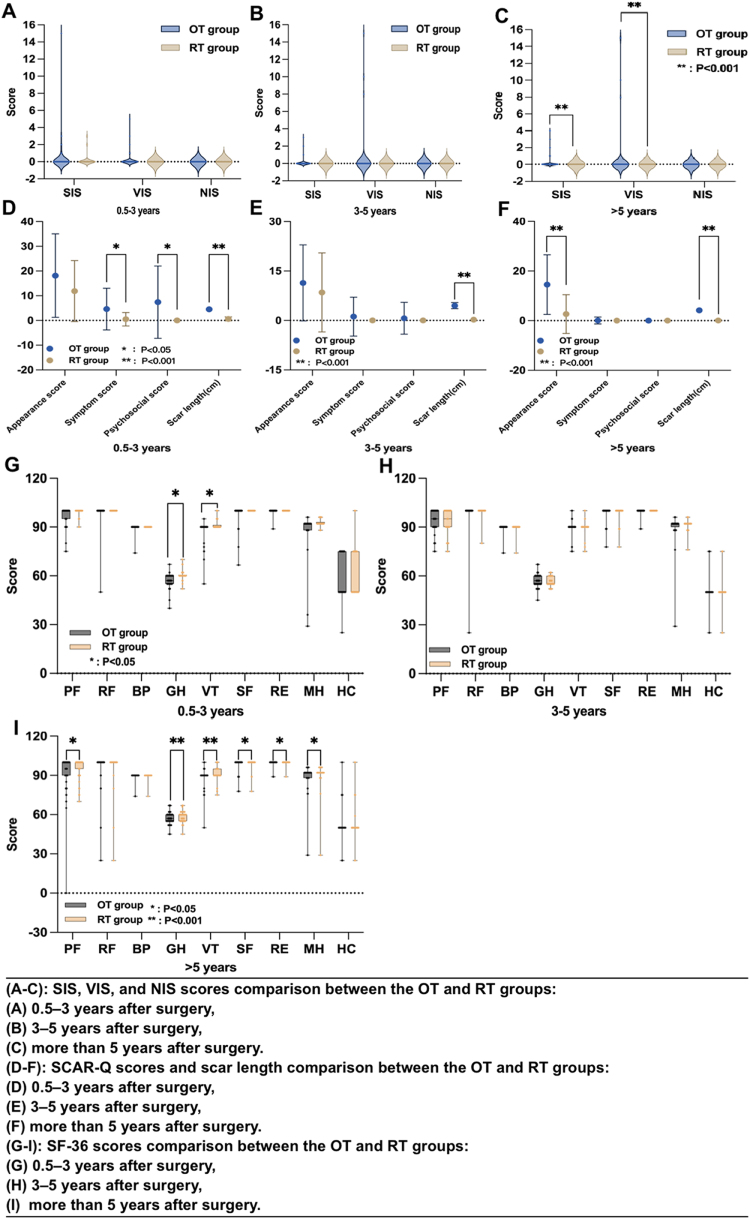
Comparison of HR-QoL outcomes over various time intervals. OT, open thyroidectomy; RT, robotic thyroidectomy; PF, physiological function; RF, role physical function; BP, body pain; GH, general health; VT, vitality; SF, social function; RE, role emotional; HC, health change; MH, mental health; SF-36, short form-36; VIS, Voice Impairment Score; SIS, Swallowing Impairment Score; NIS, Neck Impairment Score; SCAR-Q, Scar questionnaire; HR-QoL, health-related quality of life.

The RT group showed better “general health” and “vitality” at 0.5–3 years and >5 years, better “physiological function” at 0.5–3 years, and better “social function” “mental health” and “role emotional” at >5 years (*P* < 0.05). There were no statistically significant differences in QoL assessments between the two groups at postoperative intervals of 0.5–3 years, 3–5 years, or >5 years (including “physiological function,” “role physical function,” “body pain,” “social function,” “Health change,” and “role emotional”), with all *P*-values exceeding 0.05 (Fig. 2).

## Discussion

The surgical indications for benign thyroid diseases include hyperthyroidism resistant to medical therapy, high functional adenoma or causing compressive symptoms, thyroid nodules with a diameter larger than 5 cm, or thyroid enlargement due to other reasons leading to compressive symptoms[38]. Because of the rich vascularity and limited neck space, large benign thyroid nodules often present challenges in achieving adequate surgical exposure, typically necessitating the expertise of experienced surgeons. Therefore, the application of minimally invasive surgery, especially robotic thyroid surgery, for large benign thyroid nodules is relatively uncommon, and related reports are still limited. This is the first observational cohort study with long-term follow-up and large sample size to compare the safety, efficacy, and postoperative QoL between RT and OT for benign thyroid nodules, particularly those with larger tumor sizes. It provides new insights into the effectiveness of robotic surgery for benign thyroid nodules and evaluates its potential benefits in this clinical setting.

The demographic comparison of patients revealed that individuals opting for RT tended to be younger, with the average age in the RT group being lower than that in the OT group, consistent with previous studies[17]. This may be attributable to the higher aesthetic concerns regarding neck appearance in younger patients, leading to a preference for the scarless robotic surgery approach. Conversely, older patients may have lower aesthetic demands concerning neck appearance, instead opting for lower-cost open surgery. To further validate the expanded indications for RT, we conducted a stratified comparative analysis based on the mass sonographic tumor diameter. Tumors with a diameter ≥5 cm accounted for 13.33% of the cases, with the largest mass diameter being 9.3 cm in the RT group. This suggests that robotic surgery can be used for the resection of larger thyroid nodules, with broader indications than endoscopic surgery for benign thyroid disease.

Additionally, our study found that the RT group required longer operation times compared to the OT group. Several reasons account for this disparity. First, robotic surgery requires longer preoperative preparation time for the surgical instruments, with literature reporting docking times of at least 30 minutes[39]. Second, the larger volume of blood loss typically associated with large benign thyroid nodules, combined with the limited surgical access provided by robotic systems, makes bleeding control more complicated than in OT. Hemostasis is generally achieved using specialized small gauze strips, placed under camera guidance, along with energy instruments. In cases where robotic surgeries require insufflation to create operative space, using suction instruments to clear bleeding may require retracting an instrument, thereby impeding the operation and prolonging hemostasis time. Therefore, achieving hemostasis under camera guidance is more challenging in RT than in OT, requiring careful handling of each vessel entering or exiting the thyroid gland, thereby prolonging total operation time. Besides, our results demonstrate that OT is associated with significantly greater intraoperative blood loss compared to RT. This finding underscores that proficient use of energy instrument in RT can effectively manage and reduce blood loss. Furthermore, the robotic system also demonstrates distinct advantages in complex benign pathologies beyond large nodules. In our cohort, which included cases of Graves–Basedow disease – a condition marked by heightened thyroid vascularity and tissue fragility – the robotic approach proved feasible and safe.

A comparison of postoperative complications showed a significantly lower incidence of transient hypoparathyroidism in the RT group compared to the OT group, consistent with the findings of a previous prospective study[17]. This difference may be attributable to the limitations of human eye resolution in open surgery, where only traditional meticulous dissection techniques are utilized, often damaging the blood supply of A-type parathyroid glands. In contrast, the robotic surgical system provides a 3D magnified view that can more clearly identify the parathyroid glands, and the Endo-Wrist instruments (Intuitive Surgical) used in robotic surgery have flexibility beyond ergonomic features, enabling them to perform super-meticulous capsular dissection (SMCD) technology and better protect the parathyroid glands, which preserves the true capsule at the posterior aspect of the thyroid gland, thereby protecting the blood supply to the parathyroid glands. Additionally, there were no statistically significant differences between the two groups concerning other complications, such as postoperative bleeding and hoarseness. This suggests that robotic surgery may be a safer alternative compared to open surgery.

Through stratified comparative analysis of SIS, VIS, and NIS at various postoperative intervals, we found that the damage scores of SIS and VIS were significantly lower in the RT group compared to the OT group, particularly beyond 5 years postoperatively. It may be attributed to the absence of a neck incision in robotic surgery, which preserves the integrity of the platysma and better protects the fascia layer of the anterior cervical muscle during the delicate process of the free flap. Consequently, postoperative adhesions between the platysma and anterior neck muscles are reduced, thus alleviating swallowing discomfort after thyroidectomy. Additionally, the application of robotic technology increases the safety distance between energy instruments and the recurrent laryngeal nerve, thereby minimizing the risk of recurrent laryngeal nerve injury during thyroidectomy.

The comparative evaluation of HR-QoL generally revealed no significant differences between the two groups. However, RT was found to be superior to OT in the assessments of “general health,” “vitality,” “physiological function,” “social function,” “mental health,” and “role emotional” at specific time points: 0.5–3 years, 3–5 years, or beyond 5 years. We analyzed several potential reasons for this disparity. First, although postoperative scars in OT can be treated symptomatically through plastic and burn surgery or dermatology, healed incisions still negatively affect neck aesthetics and the patient’s psychological well-being. Larger neck incisions required for benign thyroid nodules, particularly for Grade III goiter[40], where the incision extends beyond the outer edge of the sternocleidomastoid muscle, have a more significant impact on neck appearance. Patients predisposed to scar formation are particularly susceptible to negative aesthetic outcomes. Previous studies have demonstrated that incision scars have a more significant negative impact on the appearance of the neck[17]. As expected, the SCAR-Q evaluation[31] demonstrated favorable cosmetic outcomes in terms of scar length and visibility in the RT group. This further demonstrates the advantages of RT in terms of cosmetic outcomes. Especially for patients with a tendency for keloid formation who require a scar-free neck, RT is undoubtedly a better surgical treatment option[41].

### Strengths and limitations

The present study had several strengths. First, it was a large-scale and long-term observational clinical study focusing on thyroidectomy for benign thyroid disease, which holds significance as a reference for minimally invasive benign thyroidectomy. Second, we conducted the first follow-up assessment of patients undergoing RT for benign tumors’ postoperative aesthetics, psychological effects, and QoL. This contributes to a more comprehensive evaluation of the effects of surgery and the overall health status of the patients. Third, this was the first report of RT for larger thyroid disease, addressing the previous limitation of minimally invasive surgery in achieving resection of large masses and providing significant empirical support for technological advancements in this field.

Nevertheless, our study had three limitations. First, our study was conducted in a single center, and surgical proficiency may vary across other centers, thus representing only the level of robotic and OT for benign nodules in our institution. Second, although our study demonstrated the feasibility of RT for resection of larger benign nodules, it did not clearly define the upper limit of nodule size as a contraindication. Third, since this was a retrospective cohort study, we could not use more accurate indicators, such as volume or weight, to reflect the size of the thyroid gland, nor could we ensure regular postoperative follow-up. Therefore, it is necessary to conduct multicenter prospective randomized controlled trials to optimize the study design, validate these findings, and enhance the quality and reliability of the results.

## Conclusion

Robotic thyroidectomy demonstrated superior cosmetic outcomes, lower complication rates, and minimal impact on postoperative QoL compared to open surgery for benign thyroid nodules, including larger ones. Therefore, with appropriate patient selection, robotic thyroidectomy may be the preferred approach for benign thyroid disease.

## Supplementary Material

## Data Availability

Datasets generated during and/or analyzed during the current study are publicly available.

## Research registration unique identifying number (UIN)

The present study is registered at www.clinicaltrials.gov (ChiCTR2400088020).

## Data sharing statement

The data supporting this study cannot be made publicly available due to restrictions in the informed consent documents and institutional policies on sensitive military medical research. Qualified researchers may contact the Ethics Committee of Army Medical University (xnyyec@sina.com) for limited access under supervision.
