# Supplementary material for: Association of long-term quality of life, safety, and efficacy: robotic vs. open thyroidectomy for benign thyroid nodules
Source: Int J Surg. 2025 Sep 24;112(1):1244–51. doi: 10.1097/JS9.0000000000003540 (PMC12825542; doi:10.1097/JS9.0000000000003540)
Supplement: Supplementary file 1 [file js9-112-1244-001.docx]

**eMethods**

**Surgical procedures**

Both surgical procedures were preceded by endotracheal intubation, general anesthesia administration, neck hyperextension, and routine surgical site disinfection and draping.

**OT:** The steps for open surgery for benign thyroid nodules, including lobectomy or total thyroidectomy, were identical to those for thyroid cancer surgery, but without lymph node dissection. We have previously detailed the OT operative procedure.

The procedure involved creating a working space, elevating a flap, making an incision in the isthmus anterior to the trachea, and incising the superior and inferior vessels using energy instruments. While addressing the posterior aspect of the thyroid gland, precise anatomical techniques were used to separate and excise the thyroid gland along the true capsule. A small portion of thyroid tissue was preserved in some cases to protect the parathyroid gland and the recurrent laryngeal nerve at its entrance into the throat. However, larger benign thyroid nodules required longer incisions and greater skin flap dissection to ensure optimal surgical exposure. Unlike thyroid cancer, large benign thyroid nodules often exhibit a rich blood supply, large upper and lower polar vessels, and significant intraoperative bleeding. Proper management of each vessel using energy-based instruments or ligation is important for maintaining a clear surgical field. Cases of inadvertent parathyroid gland removal during surgery were confirmed through intraoperative frozen sections, followed by transplantation into the ipsilateral sternocleidomastoid muscle using injection techniques.

**RT:** We have detailed the operative RT process in a previous report. Despite differences in the surgical approach and methods of field exposure, the procedural steps for RT were generally similar to those for OT. Surgical access was obtained through the unilateral axillo-breast approach or, for nodules having a diameter > 5 cm, through the bilateral axillo-breast approach. Access and skin flap elevation have previously been described in published literature. To improve thyroid exposure, suspension sutures or specialized retractors were used to retract the anterior neck muscles. Benign thyroid nodules typically exhibit high vascularity, significant isthmus thickening, and large superior and inferior vessels. Therefore, meticulous vascular handling is essential to prevent bleeding. In addition to the superior and inferior thyroid vessels, precise coagulation using ultrasonic shears or bipolar forceps is required for any blood vessels entering or exiting the thyroid gland. Using high-definition 3D visualization and enhanced dexterity of robotic surgical systems, a super-meticulous capsular dissection technique (SMCD) was used to preserve the true thyroid capsule, particularly at the posterior aspect. This allowed preservation of the parathyroid glands and recurrent laryngeal nerves.

A specialized 2-cm metal Trocar was used to cut and remove the specimen. Given the potential for tumor rupture during surgery and residual tumor following removal, the surgical cavity was thoroughly rinsed with a large volume of distilled water to prevent specimen residue.
